# The hedonic expectancy gap: a framework for understanding intention failure in precontemplation

**DOI:** 10.3389/fpsyg.2026.1789958

**Published:** 2026-04-08

**Authors:** Joseph Burge

**Affiliations:** Independent Researcher, Los Angeles, CA, United States

**Keywords:** affective forecasting, behavior initiation, experiential calibration, health behavior change, hedonic expectancy, intention formation, precontemplation, prediction error

## Abstract

This paper proposes the Hedonic Expectancy Gap, the signed discrepancy between anticipated and experienced pleasantness during behavior initiation (𝝙 = Reality - Forecast), as a proximal mechanism linking desire for change to intention formation. The purpose is to formalize a persistent ambiguity in the Transtheoretical Model: precontemplation is defined by absent intention, yet “unready” individuals are often treated in practice and in systems as lacking desire. This paper first summarizes how this practitioner- and systems-level inference has become dominant. It then synthesizes convergent evidence that many precontemplators desire change but expect initiation to feel worse than it typically does, consistent with affective-forecasting findings that forecasts are systematically inaccurate and behaviorally decisive. Finally, the paper specifies the Hedonic Expectancy Gap as a testable variable, and outlines a minimal cycle consisting of a time-locked forecast, bounded micro-test, immediate post-experience rating, 𝝙 computation, and neutral practitioner elicitation, designed to quantify hedonic prediction error and support recalibration. The Hedonic Expectancy Gap could provide a practical measurement-and-intervention scaffold for integrative health coaching and digital-health design by distinguishing expectancy-blocked non-initiation from genuine disinterest, and clarifying how affective expectation can suppress intention even when outcome value and capability are otherwise favorable.

## 1 Background

The transtheoretical model (TTM; Prochaska and Velicer, 1997) remains one of the most influential frameworks in behavioral science, describing change as progression through stages: precontemplation, contemplation, preparation, action, and maintenance. This structure provides practitioners and systems with a practical way to classify readiness and tailor intervention style, and it has become deeply embedded across health promotion, psychotherapy, and wellness coaching. However, the model’s earliest stage contains a persistent ambiguity: precontemplation is defined solely by the absence of imminent intention, not by the presence or absence of desire. The architecture, therefore, captures *when* intention is present but remains theoretically agnostic about *why* it fails to form.

Over time, a practitioner- and systems-level inference has become dominant: absence of intention is treated as absence of desire. Precontemplators are routinely described as “not ready” or “unmotivated,” which is an assumption reflected in practitioner guidance and in digital health readiness triage based on engagement proxies (Jordan, 2013; Hardcastle et al., 2015; Raihan and Cogburn, 2023; Keim-Malpass et al., 2023; Bober et al., 2024). However, empirical evidence consistently documents heterogeneity within precontemplation. Subgroups characterized by genuine desire to change but anticipated discomfort, effort, or emotional cost have been identified across behaviors (DiClemente et al., 1991; Dijkstra and de Vries, 2000; Schorr et al., 2008; Santiago-Rivas et al., 2012; Hardcastle et al., 2015). These findings suggest that early non-intention frequently reflects a friction point at initiation rather than indifference to outcomes.

A parallel body of research in affective forecasting demonstrates that anticipated emotional experience frequently diverges from experienced affect due to systematic bias (Gilbert and Wilson, 2000, 2007; Wilson and Gilbert, 2005; Meyvis et al., 2010). Forecasts are often inferred rather than simulated, rendering them vulnerable to distortion, and biased expectations can guide behavior more strongly than lived experience (Meyvis et al., 2010; Liu et al., 2022; Yang et al., 2025). Across health domains, anticipated affect predicts intention and behavior over and above cognitive determinants (Conner et al., 2015; Chitraranjan and Botenne, 2023; Yang et al., 2025). Together, previous studies have converged on two empirically supported premises: (1) early stage non-intention is heterogeneous and frequently affect-laden and (2) affective forecasts are systematically biased and behaviorally decisive.

Despite this convergence, a critical gap remains. Readiness models (e.g., TTM), intention frameworks (e.g., theory of planned behavior [TPB]; Ajzen, 1991; Ajzen and Schmidt, 2020), expectancy–value models (van der Pligt and de Vries, 1998), and self-efficacy theory (Bandura, 1997) acknowledge affective appraisal but do not isolate forecast–experience discrepancy as a measurable mechanism of intention failure. Affective forecasting research robustly documents misprediction but rarely translates forecast accuracy into a structured, repeatable intervention scaffold within health behavior change. As a result, a plausible proximal barrier of miscalibrated expectations about how initiation will feel remains theoretically recognized yet operationally diffuse.

This omission has practical significance. In applied and digital health settings, readiness is often inferred from engagement patterns or stated timelines (Raihan and Cogburn, 2023; Bober et al., 2024). When non-initiation is equated with low motivation, expectancy-blocked individuals may be misclassified. Given the evidence that anticipated affect can suppress intention even when outcome value and perceived capability are favorable (Conner et al., 2015; Yang et al., 2025), failure to detect and recalibrate hedonic misprediction may constrain the effectiveness of early stage intervention at both the individual and system levels.

The present study addresses this conceptual and methodological gap by introducing the Hedonic Expectancy Gap (HEG). The HEG is defined as the signed discrepancy between anticipated and experienced pleasantness during behavior initiation, operationalized as a measurable prediction error (Δ = Reality−Forecast), consistent with affective forecasting methodology (Gilbert and Wilson, 2000; Wilson and Gilbert, 2005; Meyvis et al., 2010; Yang et al., 2025). “Hedonic” is used in the broad valence sense (pleasant–unpleasant) to capture the experienced desirability or aversiveness of initiation, regardless of whether the anticipated cost is physical, cognitive, or social (Conner et al., 2015).

Desire for change may be present, but intention formation must pass through cognitive and affective appraisals (Ajzen, 1991; Perugini and Bagozzi, 2001). Building on recent studies that position anticipated affect as central to intention (Conner et al., 2015; Yang et al., 2025), the HEG specifies forecast accuracy as the proximal mechanism that suppresses intention, rather than anticipated affect alone. Conceptually, it reframes early non-initiation as affective miscalibration; methodologically, it proposes a minimal measurement cycle (forecast → micro-test → reality → discrepancy) to detect and recalibrate the hedonic prediction error.

By formalizing hedonic misprediction as a measurable, intervention-ready mechanism, this framework aims to clarify heterogeneity within precontemplation, refine early-stage readiness assessment, and translate descriptive findings from affective forecasting into a structured tool for use in applied health behavior change contexts.

## 2 Theoretical foundations of the hedonic expectancy gap

### 2.1 The transtheoretical model and the affective logic of early-stage change

Precontemplation, the earliest stage of the TTM, has long been recognized as internally heterogeneous (Prochaska and Velicer, 1997); however, this heterogeneity was never formalized within the model’s architecture. Contemporary analyses continue to document meaningful subtypes within precontemplation, including individuals who express a desire to change but remain stalled at initiation (Dijkstra and de Vries, 2000; Prochaska and Prochaska, 2016). Since staging defines readiness solely by stated intentions within a timeframe, the mechanisms that generate or suppress these intentions remain theoretically underspecified.

Recent applied studies have further underscored that early non-intention frequently reflects affect-laden inhibition rather than indifference (Hardcastle et al., 2015). Individuals may desire change and understand the consequences of inaction, yet they anticipate that initiating the behavior will feel unpleasant, effortful, or emotionally costly. Early subtype research gestured toward this affective logic (DiClemente et al., 1991), but the distinction remained implicit. HEG extends this line of inquiry by formalizing hedonic misprediction as a candidate proximal source of intention failure.

### 2.2 Affective forecasting and predictive bias

Affective forecasting theory provides the most direct conceptual foundation for HEG. Foundational studies established that anticipated emotional experience frequently diverges from actual experience due to systematic bias (Gilbert and Wilson, 2000; Wilson and Gilbert, 2003, 2005). Subsequent refinements have clarified recurring cognitive mechanisms underlying these distortions and demonstrated their behavioral consequences (Hoerger et al., 2010; Levine et al., 2012; Liu et al., 2022).

More recent syntheses extend this literature across clinical and health contexts, documenting both the robustness of affective misprediction and heterogeneity in how it is measured (Rizeq, 2024; Yang et al., 2025). Although forecast–experience discrepancies are increasingly indexed, they are rarely translated into structured recalibration procedures. HEG builds directly on these methodological advances by treating forecast accuracy as the mechanism of interest.

### 2.3 The theory of planned behavior and affective tolerability

In the TPB, intention arises from attitudes, subjective norms, and perceived behavioral control (Ajzen, 1991, 2015). Later refinements acknowledged affective components of attitude (Ajzen, 2002, 2011; Ajzen and Schmidt, 2020), and more recent empirical studies have highlighted anticipated affect as a central predictor of intention across health behaviors (Conner et al., 2015; Yang et al., 2025).

Despite these advancements, anticipated affect is typically embedded within broader attitudinal constructs rather than isolated as an independent determinant capable of overriding favorable cognitive evaluations. HEG reframes anticipated experiential valence as a tolerability forecast that can suppress intention even when feasibility beliefs and outcome values are favorable, aligning with contemporary expansions while specifying a measurable discrepancy mechanism.

### 2.4 Expectancy–value models and affective weighting

Expectancy–value (EV) models conceptualize behavior as a function of expected outcomes and subjective value (Feather, 1982; van der Pligt and de Vries, 1998). Later refinements acknowledged that affective evaluations influence value weighting (van der Pligt et al., 1997). Recent meta-analytic evidence indicates that anticipated affect often rivals or exceeds instrumental beliefs in predicting intention (Chitraranjan and Botenne, 2023).

However, EV formulations rarely isolate the immediately predicted felt experience of initiation as the most proximal expectation guiding action. HEG builds on this trajectory by narrowing the analytic focus to anticipated experiential valence at initiation, positioning hedonic prediction error as a determinant distinct from generalized outcome valuation.

### 2.5 Self-efficacy and affective confidence

Self-efficacy theory emphasizes the role of belief in capability in driving initiation and persistence (Bandura, 1997). Contemporary applications continue to demonstrate the predictive power of efficacy beliefs across health domains. However, capability confidence does not ensure intention when anticipated experience is perceived as aversive.

Recent affect-focused research suggests that experiential expectations can constrain the translation of efficacy into intention (Conner et al., 2015; Yang et al., 2025). HEG clarifies this boundary condition by distinguishing competence appraisal (“Can I do this?”) from affective expectancy (“Will this feel tolerable?”), integrating efficacy theory with emerging evidence on anticipated affect.

### 2.6 Cognitive load theory and anticipated effort

Cognitive load theory (CLT) explains how perceived effort is shaped by working-memory constraints and task demands (Sweller, 1988; Sweller et al., 2019). Contemporary environments characterized by frictionless design and information saturation may reduce exposure to a manageable challenge, potentially distorting perceived effort thresholds. Recent studies on stress, attentional control, and overload suggest that elevated cognitive strain amplifies anticipatory overestimation of difficulty (Eysenck et al., 2007; Sato et al., 2017). HEG integrates CLT within this broader context by treating exaggerated anticipatory discomfort as a manifestation of hedonic miscalibration under modern cognitive conditions.

### 2.7 Synthesis of theoretical foundations

Across these traditions, foundational theories have progressively incorporated affective appraisal, and recent empirical studies have strengthened the case for anticipated affect as a decisive determinant of intention. However, despite these advancements, no framework systematically isolates forecast–experience discrepancy as a measurable, recalibratable mechanism within readiness or intention models.

HEG synthesizes contemporary findings from affective forecasting, anticipated-affect research, and readiness theory by identifying the hedonic prediction error as a proximal link between desire and intention. In doing so, it extends existing models not by introducing a new affective variable, but by operationalizing forecast accuracy itself as the mechanism through which intention may be suppressed or restored.

## 3 Literature review and novelty assessment

Building on the conceptual gaps outlined in Section 2, this review evaluates whether the Hedonic Expectancy Gap represents a theoretically plausible and methodologically distinct mechanism of intention failure rather than a reformulation of existing principles. The aim is not only to assess novelty but to examine whether the hedonic prediction error satisfies three criteria required of a proximal mechanism: empirical robustness, demonstrated relevance to intention, and lack of operationalization within existing readiness frameworks.

A targeted search strategy combined database searches across PsycINFO, PubMed, Scopus, Web of Science, ScienceDirect, and APA PsycNet, supplemented by Google Scholar to capture recent and cross-disciplinary studies. Search terms included affective forecasting, anticipated affect, readiness to change, intention formation, self-efficacy, coaching, digital health engagement, hedonic expectation, prediction error, and forecast–experience discrepancy. Records were screened for relevance to affective misprediction and intention failure.

The review proceeds through three lines of inquiry:

(1) Whether affective misprediction is robust and recurrent.
(2) Whether anticipated affect reliably predicts intention yet remains under-operationalized.
(3) Whether existing readiness and behavior-change models detect or recalibrate forecast–experience discrepancy.

These criteria do not assume hedonic misprediction is the sole cause of intention failure. Identity conflict, structural constraints, and moral norms may independently inhibit action. The narrower question is whether hedonic prediction error qualifies as a distinct, measurable, and currently under-instrumented mechanism within the broader determinants of intention.

Across these domains, affective misprediction emerges as behaviorally consequential, yet within the frameworks reviewed here, forecast–experience discrepancy is not typically isolated as a primary measurement target. HEG is therefore positioned as a specific, testable integration that addresses a defined methodological gap.

### 3.1 Affective misprediction as a robust, recurrent, and under-operationalized mechanism

The strongest empirical precedent for HEG originates in affective forecasting research. Foundational work demonstrated that individuals systematically mispredict the intensity and duration of future emotional reactions (Gilbert and Wilson, 2000). Subsequent research identified recurring cognitive mechanisms underlying these distortions, positioning forecasting bias as a predictable feature of judgment rather than random error (Wilson and Gilbert, 2005; Gilbert and Wilson, 2007; Kahneman, 2011; Hoerger et al., 2010).

These errors often persist because experience does not reliably recalibrate expectations. Individuals may misremember prior forecasts as more accurate than they were (Meyvis et al., 2010), and biased expectations can predict subsequent behavior more strongly than experienced affect (Liu et al., 2022). Recent syntheses confirm the robustness of affective misprediction while noting heterogeneity in measurement approaches (Rizeq, 2024).

Although typically framed as a general decision bias, affective misprediction may be particularly relevant at the initiation of health behavior. Anticipated effort and discomfort are compressed into a scalar subjective signal that influences task engagement (Kurzban et al., 2013). When anticipated experiential cost is inflated, initiation may fail even when outcome value and perceived capability are favorable. In this context, hedonic misprediction may function as a proximal contributor to early stage intention failure.

Despite extensive documentation, affective misprediction remains minimally operationalized in applied settings. Experimental paradigms rarely pair time-locked forecasts with immediate post-behavior ratings in naturalistic contexts, nor do they embed repeated within-person recalibration cycles. Forecasting bias is well described but not routinely instrumented as an intervention-ready mechanism.

### 3.2 Anticipated affect as a reliable but fragmented predictor of intention

A substantial body of research shows that anticipated affect reliably predicts intention across domains, although its treatment remains conceptually and methodologically heterogeneous.

Within the TPB, favorable attitudes and perceived behavioral control often fail to translate into action, reflecting the well-documented intention–behavior gap. Although Ajzen (1991) acknowledged affective components of attitude, subsequent refinements retained a predominantly cognitive emphasis (Ajzen, 2002, 2011; Ajzen and Schmidt, 2020). Anticipated affect is therefore not absent from dominant models but typically embedded within broader evaluative constructs rather than isolated as a distinct mechanism.

Early critiques noted an inconsistent distinction between anticipated affect and instrumental beliefs (van der Pligt et al., 1997). Subsequent work demonstrated its independent predictive role (Bagozzi, 1992; Perugini and Bagozzi, 2001; Rivis et al., 2009; Conner et al., 2015). Meta-analytic evidence confirms anticipated affect as among the strongest predictors of intention (Chitraranjan and Botenne, 2023), while noting variability in definitions and measures. Ferrer and Ellis (2019) similarly observed that affective predictors are frequently subsumed under broader attitude constructs.

However, predictive strength does not address forecast accuracy. Most models treat anticipated affect as static and do not assess whether expectations are accurate. If anticipated affect is systematically biased, miscalibration rather than affect per se may represent a proximal mechanism of intention failure. The critical question becomes whether inaccurate affective expectations gate initiation.

Exercise psychology provides a well-characterized domain. Ruby et al. (2011) reported consistent underestimation of post-exercise positive affect relative to experienced ratings. Ekkekakis and Dafermos (2012), Loehr and Baldwin (2014), and Rhodes and Kates (2015) showed that affective judgments predict exercise behavior more strongly than instrumental beliefs. Experimental work demonstrates causal effects of anticipated affect on intention (Kwan et al., 2017), and affective experience during a single bout predicts subsequent choice (Zenko and Ekkekakis, 2019). Exercise involves immediate somatic feedback and rebound effects, which may amplify discrepancies; generalizability to other behaviors requires further study.

Across domains, anticipated affect is a robust predictor of intention, yet forecast–experience discrepancy is rarely paired, tracked within person, or embedded in recalibration procedures. HEG addresses this gap by shifting attention from anticipated affect as a predictor to forecast accuracy as a measurable and potentially correctable process.

### 3.3 Readiness models and coaching frameworks do not typically instrument hedonic expectancy

Existing readiness models do not typically operationalize hedonic prediction error.

Within the TTM, readiness is defined temporally by intention (Prochaska and DiClemente, 1983; Prochaska and Velicer, 1997), and decisional balance does not isolate anticipated affect as a distinct construct. Individuals who desire change but anticipate unpleasant experiences may therefore be grouped with those who lack desire. Practitioner materials frequently infer readiness from expressed intention (Prochaska and Norcross, 2001; Jordan, 2013; National Board for Health and Wellness Coaching, 2022). These models were not designed to assess forecasting error; the present critique is additive rather than corrective.

Empirical studies document heterogeneity within precontemplation, including expectancy-blocked subgroups (Dijkstra and de Vries, 2000; Santiago-Rivas et al., 2012; Schorr et al., 2008; Hardcastle et al., 2015). Staging algorithms operationalize readiness through intention thresholds (Hashemzadeh et al., 2019; Raihan and Cogburn, 2023), which may compress experiential barriers into a single category.

Similar scope limitations appear in organizational readiness (Shea et al., 2014; Miake-Lye et al., 2020; Gabutti et al., 2022) and behavior models such as COM-B, implementation intentions, and HAPA (Michie et al., 2011; Gollwitzer, 1999; Schwarzer and Luszczynska, 2008). These frameworks incorporate motivation and expectancies but do not isolate forecast–experience discrepancy as a measurable process.

Digital health systems often infer readiness from engagement or risk metrics (Jin, 2021; Linke, 2023) and may not distinguish indifference from hedonic miscalibration. Although Yang et al. (2025) begin by indexing forecast accuracy, relatively few interventions embed recalibration procedures.

HEG is therefore proposed as a structured method for instrumenting forecast–experience discrepancy within readiness contexts.

### 3.4 Summary: a clear and unmet need for the hedonic expectancy gap

Across forecasting research, intention models, readiness frameworks, and applied systems, affective misprediction emerges as behaviorally meaningful yet rarely measured or recalibrated in practice.

HEG is proposed as a theoretically grounded, testable integration that formalizes forecast–experience discrepancy as a measurable construct. By embedding discrepancy within a structured feedback loop, HEG seeks to examine whether hedonic misprediction functions as a modifiable contributor to early stage intention failure. Rather than offering a comprehensive account of non-initiation, HEG is advanced as a specific calibration hypothesis within broader readiness theory.

## 4 Methods of conceptual integration

Because this is a conceptual integration rather than an empirical study, “Methods” refers to the analytic steps used to consolidate existing theory and evidence into (a) a defined mechanism and (b) a minimal procedure for detecting and recalibrating hedonic prediction error (cf. Gilbert and Wilson, 2000; Kahneman, 2011; Meyvis et al., 2010). The aim is to move from descriptive evidence of forecasting bias to a structured, testable recalibration process. Four decisions structured this integration:

(1) identify the omitted mechanism,
(2) define a measurable variable,
(3) specify unbiased sampling conditions, and
(4) translate the mechanism into an applied cycle.

These steps are intended to ensure that the transition from theory to procedure is logically complete: a candidate mechanism must be specified, rendered measurable, observed under conditions that minimize distortion, and embedded within a repeatable process capable of testing and recalibrating prediction error.

Consistent with affective forecasting research, forecasting bias is treated as a structural feature of judgment rather than noise (Gilbert and Wilson, 2000; Wilson and Gilbert, 2005; Kahneman, 2011). Here, “unbiased sampling conditions” refer to procedural safeguards such as time-locking forecasts prior to behavior, using matched scales for anticipated and experienced affect, minimizing retrospective reconstruction, and preventing access to prior outcome data during forecast generation.

### 4.1 Identifying the omitted mechanism

Individuals may desire change yet withhold intention because they expect the behavior to feel unpleasant or aversive (Dijkstra and de Vries, 2000; Hardcastle et al., 2015; Santiago-Rivas et al., 2012; Schorr et al., 2008). When interpreted through affective forecasting (Gilbert and Wilson, 2000, 2007; Hoerger et al., 2010; Meyvis et al., 2010; Liu et al., 2022), some early inhibitions may be better explained as hedonic misprediction rather than low desire or capability. HEG therefore classifies a subset of pre-intention failure as hedonic prediction error: a misestimated affective cost that interrupts the transition from desire to intention (see Section 3 for empirical review).

This classification does not imply that all pre-intention failure reflects hedonic miscalibration. Alternative explanations, such as identity conflict, structural constraints, competing goals, or low perceived efficacy, may independently inhibit intention formation. Hedonic misprediction is likely most salient when outcome value and capability are favorable, desire is present, yet the anticipated experiential cost is inflated relative to subsequent experience.

### 4.2 Defining hedonic prediction error (Δ)

Hedonic prediction error was operationalized as the signed discrepancy between anticipated and experienced pleasantness: Δ = Reality−Forecast. Forecast and Reality are recorded immediately pre- and post-behavior using matched 1–10 pleasantness scales (higher scores indicate greater pleasantness). Under this orientation, Δ > 0 indicates that the experience was more pleasant than anticipated (pessimistic forecasting), Δ < 0 indicates that the experience was less pleasant than anticipated (optimistic forecasting), and |Δ| indexes misprediction magnitude regardless of direction (cf. Yang et al., 2025). Structural misprediction is well documented across forecasting studies (Gilbert and Wilson, 2000; Wilson and Gilbert, 2003; Meyvis et al., 2010; Kermer et al., 2006; Liu et al., 2022), supporting Δ as a focal mechanism rather than a secondary metric.

Although individuals may describe initiation costs in terms of effort, discomfort, or emotional burden, these appraisals are treated here as inputs to a unifying hedonic forecast, preserving semantic alignment with “hedonic.” The use of a single-item pleasantness score prioritizes parsimony and feasibility in applied contexts; however, this choice may limit internal reliability and dimensional specificity. The measure is therefore intended as a minimal calibration index rather than a comprehensive affective assessment.

#### 4.2.1 Construct anchoring: behavior-specific pleasantness definition

To prevent construct drift, “pleasantness” is anchored to a behavior-specific meaning co-defined by the client and practitioner prior to the first forecast. The practitioner elicits what “pleasantness” refers to for that behavior (e.g., for exercise initiation: physical sensations, perceived exertion, mood shift, energy, recovery, or self-consciousness) and summarizes it as a one-sentence anchor (e.g., “pleasantness = how good/bad this feels in my body and mood during and immediately after”). All subsequent Forecast and Reality ratings are made with this anchor in view, supporting interpretive consistency across trials.

The anchor may be multidimensional in description, but the recorded rating remains a single pleasantness score to preserve comparability and minimize measurement burden. This introduces subjectivity at the level of anchor definition, and cross-practitioner fidelity may vary without standardized guidance. To mitigate this risk, anchors are documented verbatim, reviewed prior to each trial, and constrained to experiential qualities during and immediately after the behavior. Further empirical testing of reliability and cross-context comparability is warranted.

### 4.3 Unbiased measurement: time-locking and immediate capture

Measurement procedures were specified to reduce retrospective reinterpretation and memory assimilation, both of which are documented components of forecasting error (Meyvis et al., 2010; Kermer et al., 2006):

- Forecast captured immediately pre-behavior, with prior Δ scores hidden.
- Reality captured immediately post-behavior on the same scale.
- Micro-tests are defined as small, bounded behavioral samples (e.g., a single brief exposure or discrete initiation attempt) to minimize emotional stakes and support interpretable comparison (cf. Sweller, 1988; Sweller et al., 2019 for load considerations; see Section 3 for empirical rationale).

These procedures are intended to reduce interpretive drift and *post*-*hoc* reconstruction so that Δ more closely reflects prediction error rather than narrative revision. They do not eliminate all potential sources of bias. Residual influences such as demand characteristics, expectancy effects, and reactivity to measurement remain possible. The goal is therefore procedural constraint rather than fully unbiased measurement, with micro-test size and timing standardized to the smallest feasible unit that captures initiation while limiting retrospective distortion.

### 4.4 Applied cycle: forecast micro-test reality delta

The mechanism was translated into a minimal sequence designed to expose and recalibrate hedonic prediction error:

- Forecast (time-locked; no prior Δ).
- Micro-Test (bounded behavioral sample).
- Reality (immediate rating).
- Delta (computed and briefly noted).

This cycle is grounded in evidence that natural experience does not consistently recalibrate hedonic expectations without explicit comparison (Meyvis et al., 2010; Liu et al., 2022) and that structured contrast between anticipated and experienced affect can support recalibration (Yang et al., 2025).

Micro-tests are deliberately bounded to maintain difficulty within a predefined tolerability range. In practice, this means selecting the smallest discrete initiation unit that meaningfully represents the target behavior (e.g., one brief exposure, one initiation attempt, or a short-duration trial) while keeping anticipated aversiveness below a threshold likely to trigger avoidance. Difficulty is therefore scaled to the individual and behavior, but constrained by duration, intensity, or exposure limits defined in advance. This approach aims to ensure the test is sufficiently representative to generate diagnostic feedback while limiting escalation of emotional stakes.

Interpretation is kept deliberately minimal to reduce narrative reconstruction effects on Δ (Wilson and Gilbert, 2005).

### 4.5 Practitioner elicitation

To preserve the independence of the Δ score, reflection is limited to neutral elicitation rather than practitioner interpretation, reducing the likelihood of suggestion that could alter how prior feelings are remembered and integrated into future forecasts (Meyvis et al., 2010). The practitioner facilitates awareness of the Δ without offering explanations beyond its direction, thereby supporting the separation of prediction error from the coached narrative.

Maintaining consistently neutral prompting may vary across practitioners. To support elicitation fidelity, guidance specifies that prompts remain descriptive rather than interpretive, avoid causal attributions, and reference only the recorded Forecast and Reality scores. Anchors and Δ values are documented verbatim, and interpretation beyond clarification of scale meaning is deferred. While some variability is likely in applied settings, the cycle’s structure constrains narrative expansion narrative expansion and centers the numerical discrepancy as the focal feedback signal.

### 4.6 Outcome logic: hedonic realism

Given that forecasting bias is systematically generated (Gilbert and Wilson, 2000; Kahneman, 2011), improvement is defined as progressive reduction of |Δ|, a movement toward more realistic expectations rather than elimination of error. Consistent with Yang et al. (2025), the objective is increased hedonic realism:

- reduced |Δ| (greater forecast accuracy in pleasantness),
- reduced systematic pessimism (mean Δ trending toward 0 when forecasts are chronically low),
- increased likelihood of intention formation as forecasts become more accurate and less aversive than expected in expectancy-blocked cases.

Reduced miscalibration does not guarantee intention formation. Contextual constraints, competing goals, identity commitments, and perceived capability may independently moderate whether improved forecast accuracy translates into action. HEG is therefore positioned as targeting a proximal calibration process that may facilitate intention under conditions where inflated experiential cost is a primary inhibitor.

Repeated positive Δ cycles may support efficacy beliefs that initiation is tolerable, thereby strengthening task-specific efficacy judgments without relying on persuasion. In this formulation, HEG does not eliminate forecasting bias but seeks to improve alignment between anticipated and experienced affect, thereby increasing the probability that desire progresses to intention in relevant cases.

## 5 Discussion and implications

### 5.1 Summary of mechanism

Evidence from precontemplation heterogeneity and affective forecasting research suggests that some instances of intention failure may reflect hedonic misprediction rather than absent desire (Dijkstra and de Vries, 2000; Gilbert and Wilson, 2000; Meyvis et al., 2010; Yang et al., 2025). Individuals may value change and believe they are capable, yet anticipate that the experience will feel more effortful, unpleasant, or emotionally taxing than it actually does. HEG formalizes this process by defining hedonic prediction error, the discrepancy between anticipated and experienced affect, as a proximal barrier to initiation in expectancy-blocked cases.

This formulation does not replace other explanations for non-initiation. Structural constraints, competing goals, identity commitments, and low perceived efficacy may independently inhibit intention formation. Rather, HEG identifies a specific calibration process that may operate alongside these factors when anticipated experiential cost is inflated. Under this interpretation, early change work may benefit from testing the forecast itself, rather than strategies aimed at strengthening motivation.

### 5.2 Δ as operational construct and instrument precursor

HEG operationalizes misprediction through a signed forecast–reality difference (Δ = Reality − Forecast) using matched scales of expected and experienced pleasantness. Prior research justifies the need for standardized procedures: forecasts are inferences rather than simulations (Gilbert and Wilson, 2000), memory distorts prior predictions (Meyvis et al., 2010), and hedonic misestimation can influence health behavior (Yang et al., 2025; Chitraranjan and Botenne, 2023). Under HEG, bias is treated as a structural property of judgment rather than contamination noise.

The delta (Δ) provides a parsimonious index of forecast–experience discrepancy that can be observed across repeated cycles. Stability and reliability of Δ trajectories over time remain empirical questions and warrant formal testing; however, repeated time-locked pairing of forecasts and experienced affect allows calibration trends to be examined within individuals. In this sense, Δ functions as a provisional calibration indicator rather than a definitive measure of accuracy.

The distinctive feature of HEG is not exposure or self-monitoring alone, both of which are well established in behavior change interventions. Rather, HEG standardizes the elicitation of anticipated and experienced valence under bias-constraining conditions and explicitly computes their discrepancy. Exposure-based approaches typically rely on experiential updating without quantifying forecast accuracy, and self-monitoring tracks behavior or affect without requiring pre-behavior predictions. HEG integrates these elements into a structured comparison process that renders calibration itself measurable and trackable across cycles.

### 5.3 Coaching and applied behavior change

From an applied perspective, HEG reframes early non-initiation not as a deficit of motivation or values, but as a potential failure of expectancy calibration. The presentation described here, of consciously valuing improved health while repeatedly failing to initiate change, has been documented in applied health contexts (Dijkstra and de Vries, 2000; Schorr et al., 2008; Hardcastle et al., 2015). When non-initiation is interpreted solely as low motivation or resistance, individuals whose avoidance is forecast-driven may be grouped with those who are genuinely indifferent (Prochaska and Norcross, 2001; Raihan and Cogburn, 2023).

When anticipated experience is miscalibrated, motivational strategies may be less effective because intention formation is constrained upstream by inaccurate forecasts of effort or unpleasantness (Conner et al., 2015; Yang et al., 2025). Under this view, calibration of anticipated experience may complement motivational and habit-based interventions by restoring the expectancy accuracy they implicitly assume, particularly in expectancy-blocked cases.

For domains where avoidance appears forecast-driven rather than value-driven, the corrective agent may be structured experience (Meyvis et al., 2010; Liu et al., 2022). The HEG cycle (time-locked forecast, bounded behavioral exposure, immediate affect rating, and Δ reflection) allows individuals to examine and quantify misprediction directly. The practitioner’s role aligns with coaching standards: elicit meaning from the discrepancy rather than persuade or advise (Jordan, 2013; National Board for Health and Wellness Coaching, 2022). When Δ trends positive across early trials, affective expectancy may recalibrate in ways that support intention. Session notes, journaling, or digital prompts can preserve Δ trajectories, offering a structured record of hedonic calibration alongside traditional indicators such as motivational language.

### 5.4 Implications: measurement and recalibration lens

Across applied settings, Δ offers a measurement-and-recalibration lens. In integrative health coaching, structured micro-tests may complement motivational strategies when desire is present, but initiation appears stalled by anticipated discomfort. Rather than relying solely on persuasion that change will feel easier than expected, the HEG cycle allows tolerability assumptions to be examined and updated through structured experience.

In organizational and digital-health environments that segment users by inferred “readiness” (see Section 3.3), Δ may serve as a potential just-in-time adaptive signal, prompting experiential micro-tests rather than delivering exclusively motivational messaging. Such applications would require consideration of feasibility, user burden, and contextual moderators, including time constraints and competing demands. Measuring forecast–experience discrepancy does not eliminate other determinants of engagement, but may offer an additional layer of precision in cases where non-engagement reflects miscalibrated expectations rather than indifference.

Across domains, Δ shifts emphasis from increasing willpower alone toward improving expectancy accuracy, clarifying one pathway through which recalibration of anticipated experience may support intention formation alongside other intervention components.

### 5.5 Future research and validation agenda

A central priority for future research is to determine whether the Hedonic Expectancy Gap (HEG) functions not only as a descriptive construct but as a correctable mechanism of intention formation. Initial studies should therefore prioritize mechanism validation over outcome optimization or downstream behavior change.

#### 5.5.1 Phase 1: naturalistic case series and mechanism verification

Early studies should employ structured case studies in applied coaching contexts, using repeated HEG cycles to examine whether hedonic prediction error behaves as a consistent, potentially learnable signal within individuals. Primary analyses would assess within-person change in forecast accuracy across repeated trials of a single target behavior, indexed by reduction in absolute error (mean |Δ|) and convergence of signed bias (mean Δ toward zero). Secondary outcomes would examine whether reductions in miscalibration co-occur with increases in task-specific self-efficacy and intention among individuals who report a desire for change but low initiation. These studies would clarify whether HEG captures a distinct expectancy-blocked presentation within precontemplation and whether calibration can occur in the absence of motivational persuasion. Attention to sample heterogeneity and the feasibility of repeated cycles will be essential at this stage.

#### 5.5.2 Phase 2: controlled trials and construct differentiation

Following preliminary mechanism verification, randomized controlled trials should compare an HEG-guided protocol with plausible active control conditions such as self-monitoring or psychoeducation, matched for contact time and behavioral exposure. Primary endpoints would include changes in hedonic expectancy calibration (|Δ|), with secondary endpoints assessing intention, self-efficacy, and behavior enactment. Analyses should test whether HEG metrics predict intention and behavior above and beyond established constructs, including perceived value, readiness, and self-efficacy, thereby evaluating discriminant and incremental validity. This phase would determine whether Δ reflects a distinct mechanism rather than an epiphenomenon of adjacent constructs.

#### 5.5.3 Phase 3: mediation and applied scaling

Later studies should formally test whether reductions in hedonic misprediction mediate improvements in self-efficacy and intention, consistent with the hypothesis that experiential calibration may complement verbal elicitation in supporting early behavior change. Additional research may explore HEG’s relevance for digital health triage systems, with attention to implementation feasibility, user burden, and contextual moderators. Demonstrating generalizability across behaviors and delivery formats would be necessary before broader applied scaling.

Across phases, HEG metrics may include signed bias (mean Δ), absolute error (mean |Δ|), and stability indices such as variability of Δ across cycles, allowing both directional and magnitude-based characterization of expectancy error. Establishing whether HEG cycles reliably reduce miscalibration and support intention formation would clarify whether hedonic expectancy calibration represents a measurable and potentially intervention-ready mechanism rather than a diffuse motivational construct.

## 6 Conclusion

HEG refines existing readiness and intention frameworks by formalizing hedonic misprediction as a measurable and potentially correctable mechanism. Δ quantifies the gap between anticipated and experienced affect, and the HEG cycle offers a structured method for examining and, where possible, narrowing that gap through iterative tests.

If empirically validated, HEG may provide a measurement scaffold and an applied procedure for addressing a recurrent barrier identified across affective forecasting and health-behavior research: the systematic misestimation of how effortful or unpleasant initiation will feel. Within this perspective, sustainable change may depend not only on motivational intensity but also on the accuracy of experiential expectation. HEG is therefore proposed as a testable refinement of existing frameworks, inviting further empirical evaluation rather than replacing established models.

## References

[ref1] Ajzen I. (1991). The theory of planned behavior. Organ. Behav. Hum. Decis. Process. 50, 179–211.

[ref2] Ajzen I. (2002). Perceived behavioral control, self-efficacy, locus of control, and the theory of planned behavior. J. Appl. Soc. Psychol. 32, 665–683. doi: 10.1111/j.1559-1816.2002.tb00236.x

[ref3] Ajzen I. (2011). The theory of planned behaviour: reactions and reflections. Psychol. Health 26, 1113–1127. doi: 10.1080/08870446.2011.613995, 21929476

[ref4] Ajzen I. (2015). Consumer attitudes and behavior: the theory of planned behavior applied to food consumption decisions. Riv. Econ. Agrar. 70, 121–138. doi: 10.13128/REA-18003

[ref5] Ajzen I. Schmidt P. (2020). “Changing behavior using the theory of planned behavior,” in The Handbook of Behavior Change, ed. Hagger M. S. (Cambridge: Cambridge University Press), 17–31.

[ref6] Bagozzi R. P. (1992). The self-regulation of attitudes, intentions, and behavior. Soc. Psychol. Q. 55, 178–204.

[ref7] Bandura A. (1997). Self-Efficacy: The Exercise of Control. New York, NY: W. H. Freeman.

[ref8] Bober T. Courtenay M. Hernando M. E. Lee S. H. Litchfield I. Marston H. . (2024). Digital health readiness: making digital health care more inclusive. JMIR Mhealth Uhealth 12:e58035. doi: 10.2196/58035, 39383524 PMC11499716

[ref9] Chitraranjan C. Botenne C. (2023). Association between anticipated affect and behavioral intention: a meta-analysis. Curr. Psychol. 43, 1929–1942. doi: 10.1007/s12144-023-04383-w, 37359634 PMC9981260

[ref10] Conner M. McEachan R. Taylor N. O'Hara J. Lawton R. (2015). Role of affective attitudes and anticipated affective reactions in predicting health behaviors. Health Psychol. 34, 642–652. doi: 10.1037/hea0000143, 25222083

[ref11] DiClemente C. C. Prochaska J. O. Fairhurst S. K. Velicer W. F. Velasquez M. M. Rossi J. S. (1991). The process of smoking cessation: an analysis of precontemplation, contemplation, and preparation stages of change. J. Consult. Clin. Psychol. 59, 295–304. 2030191 10.1037//0022-006x.59.2.295

[ref12] Dijkstra A. de Vries H. (2000). Subtypes of precontemplating smokers defined by different long-term plans to change their smoking behavior. Health Educ. Res. 15, 423–434. 11066460 10.1093/her/15.4.423

[ref13] Ekkekakis P. Dafermos M. (2012). “Exercise is a many-splendored thing…,” in The Oxford Handbook of Exercise Psychology, ed. Acevedo E. O. (New York, NY: Oxford University Press), 295–333.

[ref14] Eysenck M. W. Derakshan N. Santos R. Calvo M. G. (2007). Anxiety and cognitive performance: attentional control theory. Emotion 7, 336–353. doi: 10.1037/1528-3542.7.2.336, 17516812

[ref15] Feather N. T. (1982). “Expectancy-value approaches: present status and future directions,” in Expectations and Actions: Expectancy-Value Models in Psychology, ed. Feather N. T. (Hillsdale, NJ: Erlbaum), 395–396.

[ref16] Ferrer R. A. Ellis E. M. (2019). Moving beyond categorization to understand affective influences on real world health decisions. Soc. Personal. Psychol. Compass 13:e12502. doi: 10.1111/spc3.12502, 33912229 PMC8078832

[ref17] Gabutti I. Colizzi C. Sanna T. (2022). Assessing organizational readiness to change through a framework applied to hospitals. Public Organ. Rev. 23, 1–22. doi: 10.1007/s11115-022-00628-7

[ref18] Gilbert D. T. Wilson T. D. (2000). “Miswanting: some problems in the forecasting of future affective states,” in Feeling and Thinking: The Role of Affect in Social Cognition, ed. Forgas J. P. (Cambridge: Cambridge University Press), 178–197.

[ref19] Gilbert D. T. Wilson T. D. (2007). Prospection: experiencing the future. Science 317, 1351–1354. doi: 10.1126/science.1144161, 17823345

[ref20] Gollwitzer P. M. (1999). Implementation intentions. Am. Psychol. 54, 493–503.

[ref21] Hardcastle S. J. Hancox J. Hattar A. Maxwell-Smith C. Thøgersen-Ntoumani C. Hagger M. S. (2015). Motivating the unmotivated: how can health behavior be changed in those unwilling to change? Front. Psychol. 6:835. doi: 10.3389/fpsyg.2015.00835, 26136716 PMC4468355

[ref22] Hashemzadeh M. Rahimi A. Zare-Farashbandi F. Alavi-Naeini A. M. Daei A. (2019). Transtheoretical model of health behavioral change: a systematic review. Iran. J. Nurs. Midwifery Res. 24, 83–90. doi: 10.4103/ijnmr.IJNMR_94_17, 30820217 PMC6390443

[ref23] Hoerger M. Quirk S. W. Lucas R. E. Carr T. H. (2010). Cognitive determinants of affective forecasting errors. Judgm. Decis. Mak. 5, 365–373. doi: 10.1017/S1930297500002163, 21912580 PMC3170528

[ref24] Jin E. (2021). How to influence patient health behaviors: Virtual care opportunities. HTD Health. Available online at: https://htdhealth.com/insights/how-to-influence-patient-health-behaviors-virtual-care-opportunities/ (Accessed February 25, 2026).

[ref25] Jordan M. (2013). How to be a Health Coach: An Integrative Wellness Approach. San Rafael, CA: Global Medicine Enterprises.

[ref26] Kahneman D. (2011). Thinking, Fast and Slow. New York, NY: Farrar, Straus and Giroux.

[ref27] Keim-Malpass J. Clark M. T. Shill J. Moorman J. R. Lake D. E. (2023). Beyond prediction…. Learn. Health Syst. 7:e10323. doi: 10.1002/lrh2.10323, 36654806 PMC9835046

[ref28] Kermer D. A. Driver-Linn E. Wilson T. D. Gilbert D. T. (2006). Loss aversion is an affective forecasting error. Psychol. Sci. 17, 649–653. doi: 10.1111/j.1467-9280.2006.01760.x, 16913944

[ref9001] Kurzban R. Duckworth A. Kable J. W. Myers J. (2013). An opportunity cost model of subjective effort and task performance. Behavioral and Brain Sciences. 36, 661–679. doi: 10.1017/S0140525X12003196 24304775 PMC3856320

[ref29] Kwan B. M. Stevens C. J. Bryan A. D. (2017). What to expect when you’re exercising: an experimental test of the anticipated affect–exercise relationship. Health Psychol. 36, 309–319. doi: 10.1037/hea0000453, 27991804 PMC6317525

[ref30] Levine L. J. Lench H. C. Kaplan R. L. Safer M. A. (2012). Accuracy and artifact: reexamining the intensity bias in affective forecasting. J. Pers. Soc. Psychol. 103, 584–605. doi: 10.1037/a0029544, 22889075

[ref31] Linke S. (2023). Leveraging GenAI to optimize behavior change predictions. Omada Health. Available online at: https://www.omadahealth.com/resource-center/leveraging-genai-to-optimize-behavior-change-predictions (Accessed February 25, 2026).

[ref32] Liu L. Wunun S. Fang P. Jiang Y. Tian L. (2022). Be optimistic or be cautious? Affective forecasting bias in allocation decisions and its effect. Front. Psychol. 13:1026557. doi: 10.3389/fpsyg.2022.1026557, 36582312 PMC9793408

[ref33] Loehr V. G. Baldwin A. S. (2014). Affective forecasting error in exercise: differences between physically active and inactive individuals. Sport Exerc. Perform. Psychol. 3, 177–183. doi: 10.1037/spy0000006

[ref34] Meyvis T. Ratner R. K. Levav J. (2010). Why don't we learn to accurately forecast feelings? How misremembering our predictions blinds us to past forecasting errors. J. Exp. Psychol. Gen. 139, 579–589. doi: 10.1037/a0020285, 20853995

[ref35] Miake-Lye I. M. Delevan D. M. Ganz D. A. Mittman B. S. Finley E. P. (2020). Unpacking organizational readiness for change: an updated systematic review and content analysis of assessments. BMC Health Serv. Res. 20:106. doi: 10.1186/s12913-020-4926-z, 32046708 PMC7014613

[ref36] Michie S. van Stralen M. M. West R. (2011). The behaviour change wheel: a new method for characterising and designing behaviour change interventions. Implement. Sci. 6:42. doi: 10.1186/1748-5908-6-42, 21513547 PMC3096582

[ref37] National Board for Health and Wellness Coaching (2022) Health and Wellness Coach certifying exam: Content outline [PDF]. National Board for Health & Wellness Coaching. Available online at: https://www.nbme.org/sites/default/files/2022-05/NBHWC_Content_Outline.pdf (Accessed February 25, 2026).

[ref38] Perugini M. Bagozzi R. P. (2001). The role of desires and anticipated emotions in goal-directed behaviours: broadening and deepening the theory of planned behaviour. Br. J. Soc. Psychol. 40, 79–98. doi: 10.1348/014466601164704, 11329835

[ref39] Prochaska J. O. DiClemente C. C. (1983). Stages and processes of self-change of smoking: toward an integrative model of change. J. Consult. Clin. Psychol. 51, 390–395. 6863699 10.1037//0022-006x.51.3.390

[ref40] Prochaska J. O. Norcross J. C. (2001). Stages of change. Psychother. Theory Res. Pract. Train. 38, 443–448. doi: 10.1037/0033-3204.38.4.443

[ref41] Prochaska J. O. Prochaska J. M. (2016). Changing to Thrive. Center City, MN: Hazelden Publishing.

[ref42] Prochaska J. O. Velicer W. F. (1997). The transtheoretical model of health behavior change. Am. J. Health Promot. 12, 38–48. 10170434 10.4278/0890-1171-12.1.38

[ref43] Raihan N. Cogburn M. (2023). Stages of Change Theory. In StatPearls. Available online at: https://www.ncbi.nlm.nih.gov/books/NBK556005/ (Accessed February 25, 2026). 32310465

[ref44] Rhodes R. E. Kates A. (2015). Can the affective response to exercise predict future motives and physical activity behavior? A systematic review of published evidence. Ann. Behav. Med. 49, 715–731. doi: 10.1007/s12160-015-9704-5, 25921307

[ref45] Rivis A. Sheeran P. Armitage C. J. (2009). Expanding the affective and normative components of the theory of planned behavior: a meta-analysis of anticipated affect and moral norms. J. Appl. Soc. Psychol. 39, 2985–3019. doi: 10.1111/j.1559-1816.2009.00558.x

[ref46] Rizeq J. (2024). Affective forecasting and psychopathology: a scoping review. Clin. Psychol. Rev. 108:102392. doi: 10.1016/j.cpr.2024.102392, 38244480

[ref47] Ruby M. B. Dunn E. W. Perrino A. Gillis R. Viel S. (2011). The invisible benefits of exercise. Health Psychol. 30, 67–74. doi: 10.1037/a0021859, 21299296

[ref48] Santiago-Rivas M. Velicer W. F. Redding C. A. Prochaska J. O. Paiva A. L. (2012). Cluster subtypes within the precontemplation stage of change for sun protection behavior. Psychol. Health Med. 17, 311–322. doi: 10.1080/13548506.2011.630401, 22175661 PMC3310972

[ref49] Sato R. A. Drennan J. Lings I. (2017). Problem recognition: integrating help-seeking theory in social marketing. J. Soc. Mark. 7, 2–17. doi: 10.1108/JSOCM-06-2015-0034

[ref50] Schorr G. Ulbricht S. Schmidt C. O. Baumeister S. E. Rüge J. Schumann A. . (2008). Does precontemplation represent a homogeneous stage category? A latent class analysis on German smokers. J. Consult. Clin. Psychol. 76, 840–851. doi: 10.1037/a0013037, 18837601

[ref51] Schwarzer R. Luszczynska A. (2008). How to overcome health-compromising behaviors: the health action process approach. Eur. Psychol. 13, 141–151. doi: 10.1027/1016-9040.13.2.141

[ref52] Shea C. M. Jacobs S. R. Esserman D. A. Bruce K. Weiner B. J. (2014). Organizational readiness for implementing change: a psychometric assessment of a new measure. Implement. Sci. 9:7. doi: 10.1186/1748-5908-9-7, 24410955 PMC3904699

[ref53] Sweller J. (1988). Cognitive load during problem solving: effects on learning. Cogn. Sci. 12, 257–285.

[ref54] Sweller J. van Merriënboer J. J. G. Paas F. (2019). Cognitive architecture and instructional design: 20 years later. Educ. Psychol. Rev. 31, 261–292. doi: 10.1007/s10648-019-09465-5

[ref55] van der Pligt J. de Vries N. K. (1998). Belief importance in expectancy-value models of attitudes. J. Appl. Soc. Psychol. 28, 1339–1354.

[ref56] van der Pligt J. Zeelenberg M. van Dijk W. W. de Vries N. K. Richard R. (1997). Affect, attitudes and decisions: let’s be more specific. Eur. Rev. Soc. Psychol. 8, 33–66.

[ref57] Wilson T. D. Gilbert D. T. (2003). “Affective forecasting,” in Advances in Experimental Social Psychology, ed. Zanna M. P., vol. 35 (San Diego, CA: Elsevier Academic Press), 345–411.

[ref58] Wilson T. D. Gilbert D. T. (2005). Affective forecasting: knowing what to want. Curr. Dir. Psychol. Sci. 14, 131–134. doi: 10.1111/j.0963-7214.2005.00355.x

[ref59] Yang M. Z. Conner M. Sheeran P. (2025). Foreseeing versus feeling: how accuracy of affective forecasting relates to health behavior change. Ann. Behav. Med. 59:kaaf084. doi: 10.1093/abm/kaaf084, 41177145

[ref60] Zenko Z. Ekkekakis P. (2019). Internal consistency and validity of measures of automatic exercise associations. Psychol. Sport Exerc. 43, 4–15. doi: 10.1016/j.psychsport.2018.12.005

